# The emerging landscape of performance-enhancing peptides modulating GH-IGF1 axis: bridging the gap between clinical evidence and patient self-administration

**DOI:** 10.3389/fendo.2026.1822475

**Published:** 2026-06-18

**Authors:** Aleksander Dominikowski, Zofia Rękoś, Michał Olejarz, Ewelina Szczepanek-Parulska, Remigiusz Domin, Marek RuchaŁa

**Affiliations:** 1 Students’ Research Group at the Department of Endocrinology, Metabolism and Internal Medicine, Poznan University of Medical Sciences, Poznan, Poland; 2 Department of Endocrinology, Metabolism and Internal Medicine, Poznan University of Medical Sciences, Poznan, Poland

**Keywords:** endocrinology, growth hormone secretagogues, IGF-1 analogues, off-label use, peptide hormones, performance-enhancing drugs, sports doping

## Abstract

Performance-enhancing drugs (PEDs) marketed as “research compounds” include unregulated peptides intended to modulate the growth hormone–insulin-like growth factor-1 (GH–IGF-1) axis. The agents most commonly encountered in clinical practice and online self-administration protocols include growth hormone-releasing hormone (GHRH) analogues (e.g., sermorelin, tesamorelin, CJC-1295 with Drug Affinity Complex [DAC], CJC-1295 without DAC), growth hormone secretagogues (GHS; e.g., growth hormone-releasing peptide-2 (GHRP-2), growth hormone-releasing peptide-6 (GHRP-6), hexarelin, ipamorelin), the growth hormone (GH) fragment - AOD9604 (hGH 176–191), and insulin-like growth factor-1 (IGF-1) analogues (e.g., pegylated mechano growth factor (PEG-MGF), IGF-1 Long R3 (IGF-1 LR3)). Reported adverse effects span endocrine and metabolic disturbances (including prolactin and cortisol elevations, appetite changes, and dysglycaemia), fluid retention syndromes, musculoskeletal symptoms (myalgia/arthralgia), and injection-site reactions. Given the absence of regulatory approval for physique- or performance-related indications and the uncertainty surrounding product composition, dose, and stacking practices in unregulated supply chains, clinicians increasingly require a pragmatic framework to interpret symptoms and laboratory abnormalities in patients using these compounds. This narrative review contrasts peer-reviewed pharmacokinetic/pharmacodynamic and clinical evidence with commonly encountered online self-administration protocols, stratifying peptides into evidence tiers from regulatory-grade randomized trial data to a complete absence of human studies, and highlights the resulting uncertainty around putative performance and recomposition benefits. We summarise structural characteristics, pharmacologic effects, and commonly reported dosing patterns, and we synthesise clinically relevant adverse effects with particular attention to hormonal imbalance, endocrine–metabolic risk, and biologically plausible but unproven mitogenic concerns. Finally, we propose a clinically oriented assessment algorithm to support exposure history taking, triage of symptom domains, and risk communication without legitimising off-label peptide regimens.

## 1 Introduction

Across fitness culture, aesthetic goals and performance optimisation increasingly motivate training alongside health-related concerns. Consistent with this trend, in a 2017 European survey, body-related motives such as weight control or appearance were reported by a substantial proportion of respondents, whereas health and general fitness were the most frequently cited overall motivations for engaging in physical activity (1). In parallel, the use of over-the-counter (OTC) substances marketed for performance enhancement appears common - a 2023 study across eight European countries reported a 10.4% prevalence of such use (2). For a subset of individuals, pursuit of an idealised physique becomes compulsive and is associated with muscle dysmorphia. Performance-enhancing drug (PED) use, including anabolic-androgenic steroids (AAS) is associated with higher muscle dysmorphia symptoms severity, compared with non-users (3). This psychological driver often leads patients to bypass clinical oversight in favour of unregulated digital resources. The adverse effects of AAS are well described, spanning cardiovascular, hepatic, endocrine/sexual, haematological, and psychiatric domains (4–12).

The use of exogenous growth hormone (GH) for physique- and performance-oriented purposes is not a new phenomenon (13). Historical analyses of doping indicate that GH misuse likely emerged by the early 1980s and increased further with the availability of recombinant GH (rGH), often driven by perceived anabolic and lipolytic effects despite limited proof of meaningful performance gains. Meta-analyses and randomized trials indicate that while GH administration increases lean body mass, this is predominantly attributable to extracellular fluid retention rather than muscle hypertrophy. Consequently, rGH does not confer significant benefits in strength or aerobic power in healthy adults (14). Controlled trials in healthy adults, including physically fit individuals, suggest that GH may increase lean body mass but does not reliably improve strength or exercise capacity and is associated with a higher rate of adverse effects such as edema and fatigue. In practice, “GH doping” is also discussed in a broader sense as manipulation of the same endocrine axis using GH secretagogues and IGF-1/IGF-1 analogues - substances that are explicitly listed as prohibited under the World Anti-Doping Agency (WADA) Prohibited List (14–17).

Against this backdrop, interest has expanded toward peptide-based compounds promoted as “safer” alternatives for improving recovery, fat loss, and anabolism. In this review, we use the term performance-enhancing peptides (PEPs) to describe peptide-based agents used off-label for physique and performance purposes, targeting the growth hormone–insulin-like growth factor-1 (GH–IGF-1) axis. Many PEPs are derivatives of GH or modulators of the GH–IGF-1 axis. GH is a 191–amino acid pituitary peptide hormone released in a pulsatile, sleep-linked manner under hypothalamic control (18–22). At the cellular level, GH binding to the GH receptor activates Janus Kinase-2 (JAK2)/STAT signalling and promotes IGF-1 production, while secretion is influenced by factors such as exercise, hypoglycaemia, fasting, obesity, and ageing (23–28).

More broadly, the success of incretin-based therapies for obesity and diabetes has likely increased public familiarity with peptide injectables, potentially lowering barriers to off-label peptide experimentation (29–31). Aims of this narrative review are: (I) map the main GH–IGF-1–modulating peptides used off-label and summarise PK/PD and human evidence; (II) synthesise endocrine–metabolic adverse effects relevant to clinical encounters; (III) compare clinical evidence with patient self-administration protocols (grey literature) and translate this into counselling/risk interpretation. The intent is to support endocrinologists and other clinicians in recognising potential harms, counselling patients, and framing risk in the absence of robust long-term data.

## 2 Literature search strategy

This article was conducted as a narrative review because the available evidence on GH–IGF-1-axis PEPs is highly heterogeneous with respect to compounds, populations, endpoints, and study design, and because a quantitative synthesis would have been inappropriate for most agents included in the review.​ The literature search was performed in PubMed, Google Scholar, and the Cochrane Library for publications from January 1989 to January 2026, restricted to English-language sources.​ Search terms combined individual compound names (e.g., “tesamorelin”, “CJC-1295”, “GHRP-2”, “hexarelin”, “ipamorelin”, “AOD9604”, “PEG-MGF”, “IGF-1 LR3”) with class-level terms such as “growth hormone secretagogue”, “GHRP”, “GHRH analogue”, “growth hormone releasing hormone analogue”, “IGF-1 analogue”, “growth hormone”, “IGF-1”, “adverse effects”, “body composition”, “hypertrophy”, “doping” and “sports”. ​Compounds were selected for inclusion if they fulfilled at least one of the following criteria: (I) peer-reviewed human pharmacokinetic, pharmacodynamic, or clinical literature existed; (II) the compound was repeatedly identified in gray literature as commonly self-administered in physique- or performance-oriented settings; or (III) the compound had sufficient regulatory, pharmacologic, or toxicologic relevance to clinical encounters despite sparse direct human evidence.​ Gray literature sources, including commercial peptide websites, bodybuilding-oriented blogs, and large public discussion forums, as Reddit, one of the world’s largest publicly accessible discussion forums, were searched separately using structured compound-specific queries to identify substances and self-administration patterns encountered by patients outside medical supervision.​ These sources were treated as anthropological and behavioral material documenting real-world narratives, dosing beliefs, and stacking practices; they were not used as primary evidence of efficacy or safety and must not be interpreted as clinical guidance.

Because the review was narrative in design, no formal risk-of-bias scoring tool or GRADE framework was applied.​ Instead, the synthesis prioritised peer-reviewed human data where available, used preclinical studies only to provide biologic context, and explicitly distinguished compounds supported by randomized trials or phase I PK/PD studies from those supported mainly by preclinical or anecdotal evidence.​ To support transparency of narrative-review quality, compliance with the SANRA tool was self-assessed by the authors across the six SANRA domains; the full domain-by-domain assessment with brief justifications and pointers to the relevant manuscript sections is provided as **Supplementary Table 1** (32).

## 3 Discussion

### 3.1 Important properties of the GH–IGF-1 axis in shaping physique

The somatotropic (GH–IGF−1) axis influences body composition through coordinated effects on whole−body protein metabolism, substrate utilisation, and, under specific conditions, skeletal muscle remodelling. Mechanistically, GH actions in muscle can be conceptualised as IGF−1–dependent effects, mediated by systemic and/or locally produced IGF−1, and IGF−1–independent effects, reflecting direct GH receptor signalling within myofibres.

Experimental models provide evidence for direct, IGF−1–independent GH signalling, although its quantitative contribution to human hypertrophy remains uncertain (33–35). In bovine muscle cells, GH stimulates protein synthesis without upregulating IGF−1 mRNA, whereas IGF−1 exerts a stronger net anabolic effect by simultaneously stimulating protein synthesis and suppressing degradation; at higher concentrations it also promotes myoblast proliferation (33). In mouse models with muscle−specific disruption of GH or IGF−1 receptor signalling, GH can modulate muscle metabolism even when IGF−1 receptor signalling is impaired, but these data do not establish clinically meaningful GH−driven hypertrophy per se (34, 35).

By contrast, the IGF−1−dependent pathway is well characterised and is generally considered central to hypertrophic signalling. IGF−1 binding to IGF−1 receptor (IGF−1R) activates insulin receptor substrate−1 (IRS−1), phosphoinositide 3−kinase (PI3K), and downstream Akt, with subsequent engagement of mTOR signalling; these effects are modulated by amino acid availability (36–39). IGF−1 can also attenuate catabolic pathways by suppressing FoxO−mediated transcription and components of the ubiquitin–proteasome system, and intersects with myostatin pathways that negatively regulate muscle mass (40–44). Overall, IGF−1 provides a more consistently documented driver of skeletal-muscle hypertrophy than GH itself, with GH contributing permissive and context-dependent effects.

### 3.2 Lipolytic effect of GH

Beyond its hypertrophic actions on skeletal muscle, growth hormone is frequently used in physique−oriented settings because of its perceived ability to reduce adipose tissue and increase lean mass. Mechanistically, GH promotes lipolysis, particularly in visceral adipose tissue (VAT) by stimulating triglyceride breakdown and increasing circulating free fatty acids (FFA), largely via activation of hormone−sensitive lipase (HSL) and related triglyceride hydrolases, and by suppressing anti−lipolytic signals (45–48). GH can also inhibit lipoprotein lipase (LPL), thereby reducing fatty-acid uptake into adipose tissue and limiting re−esterification and storage of lipid droplets (49).

These molecular effects are consistent with human data. In growth hormone−deficient patients, controlled GH administration has been associated with shifts in substrate utilisation towards greater lipid oxidation on indirect calorimetry (50). In states of GH excess, including acromegaly and controlled GH infusion studies, clinical data demonstrate robust stimulation of adipose tissue lipolysis, with elevated FFA, reduced fat mass, and associated alterations in insulin sensitivity and lipid metabolism (51–53). In a physique−oriented interpretation, increased FFA availability and enhanced lipid oxidation are often viewed as supportive of fat−loss goals, particularly when combined with training, although these shifts may carry clinically relevant downstream endocrine and metabolic consequences (54).

### 3.3 Tissue regeneration and “anti-aging”

Exogenous growth hormone has long been promoted, both clinically and off−label, for its potential to support tissue repair and recovery. In paediatric populations with severe burns, recombinant GH may shorten healing time and reduce hospital stay, whereas a comparable randomised trial in severely burned adults did not demonstrate a significant reduction in wound−healing time (55, 56). A broader review of randomised studies reported more optimistic signals overall but highlighted major limitations, including variable study quality and insufficient power, which preclude firm conclusions regarding efficacy in adult wound healing (57). Collectively, available data support biological plausibility but do not define a reliable, clinically generalisable benefit for tissue repair in adults.

Within the musculoskeletal system, the GH–IGF−1 axis is implicated in connective−tissue remodelling, often emphasised in training contexts where tendon, ligament, and extracellular matrix (ECM) adaptation may influence injury risk and recovery (58–60). GH can stimulate type I collagen synthesis in tendon and skeletal muscle, and IGF−1–mediated pathways support ECM synthesis and cellular proliferation during repair, providing a mechanistic basis for perceived recovery benefits (58–60). However, the magnitude and clinical relevance of such effects in healthy athletes remain uncertain.

Claims that GH confers a direct “anti−aging” effect are substantially more contentious. Human data linking GH−axis manipulation to delayed metabolic ageing or improved long−term health outcomes remain inconclusive, whereas animal−model literature describes complex trade−offs in GH/IGF−1 signalling, including lifespan extension and tumour modulation, that do not translate straightforwardly into clinical recommendations (61, 62). Somatotropic status also appears coupled to inflammatory tone: in severe obesity, restoration of the GH/IGF−1 axis after weight loss is associated with lower CRP and improved body composition, while pro−inflammatory cytokines can impair GH receptor signalling and promote functional GH resistance (63–65). Overall, what is often described as an “anti−aging” effect is more plausibly interpreted as improved tissue repair capacity and functional recovery in specific contexts, rather than a proven ability to slow intrinsic ageing biology (58, 59).

### 3.4 Peptides - overview

This section summarises the peptide compounds most commonly encountered in off-label use among athletes, bodybuilders, and recreational users, particularly analogues and secretagogues that act on the GH-IGF-1 axis. These agents are typically used with the aim of altering body composition and supporting perceived “recovery” or performance outcomes. At the same time, increasing clinical concern relates to safety, regulation, and product quality. Many of these peptides circulate outside standard medical oversight and are frequently marketed online or through informal channels as “for research purposes only” (“research chemicals”), with uncertain purity, dose consistency, and sterility. This context makes it essential to present a clear, clinically oriented overview of what is being used, what is known from human evidence, and what risks may be relevant in practice.

The peptides most widely discussed and used can be grouped into four broad categories: (I) GHRH analogues, (II) GH peptide analogues/GH-derived fragment, (III) GH secretagogues, and (IV) IGF-1 analogues.

For rapid orientation, **Table 1** summarises the mechanisms, pharmacokinetics, key safety signals, and the strength of the human evidence base for each compound (including an evidence tier and a brief description of human data). Detailed subsections highlight clinically relevant nuances, limitations of the human evidence, and practical implications for endocrinology.

**Table 1:** Growth hormone/IGF-1–axis–related peptides and secretagogues used off-label for body composition and performance: administration, mechanism, off-label claims, key safety signals, and strength of the human evidence base.

Compound	Route & Pharmacokinetics	Primary biological effect	Purported off-label rationale	Key safety signals / concerns	Tier	Brief description of the human evidence base	
GHRH analogues							
Sermorelin (GRF 1–29/GHRH 1-29)	i.v./s.c (66, 93); short-acting t½ (as disappearance half-time)~4,3+/-1,4 min (118)	↑ endogenous pulsatile GH secretion → secondary ↑ IGF-1 (119–121)	“Body recomposition” (fat loss, muscle gain); Enhanced testicular LH/hCG receptor abundance and steroidogenic responsiveness to hCG (GH/IGF-I-mediated)(animal data) (69, 93)	Injection-site pain; short-term flushing, nausea, dizziness, headache; rare allergic reaction; transient PRL rise reported (small/rapid) (66, 93) Mitogenic GH/IGF-1 signalling (theoretical oncologic concern); no established clinical carcinogenic signal (117). No long term safety data available.	A*	Historical regulatory approval (FDA approval for paediatric GHD historically held; commercially withdrawn 2008); phase I/II human studies in adult endocrine testing; no current regulatory approval for performance/body-composition indications (66–68, 118–121).	
CJC-1295 (with DAC)	s.c (80).; albumin-binding DAC; t½ ~5.8–8 days (in healthy adults) (80)	Sustained ↑ endogenous GH secretion → consequent ↑ IGF-1 (80)	Potential metabolic/anabolic benefits through sustained GH/IGF−1 elevation, including favourable shifts in body composition (e.g., increased lean mass, reduced adiposity) and enhanced metabolic signaling (80, 122)	Generally well−tolerated in short−term human studies; no serious adverse events reported at tested doses; mild local and systemic effects related to GH axis stimulation have been observed (80). Mitogenic GH/IGF-1 signalling (theoretical oncologic concern); no established clinical carcinogenic signal (117). No long term safety data available.	B	Phase I PK/PD studies in healthy adults; no controlled efficacy data (80, 122).	
CJC-1295 (without DAC) (Mod GRF 1–29/Mod GHRH 1-29)	No peer-reviewed clinical studies in humans have reported the route of administration; t½ not reported in human studies	↑ endogenous, pulsatile GH release → transient ↑ GH, and downstream ↑ IGF−1 (when integrated over repeated dosing patterns) (inferred from GRF 1–29 study) (121)	No peer-reviewed human studies.	No peer-reviewed human studies. Mitogenic GH/IGF-1 signalling (theoretical oncologic concern); no established clinical carcinogenic signal (117).	D	No peer-reviewed human studies.	
Tesamorelin	s.c (76).; N-terminal hexenoyl moiety → improved stability t½~26–38 min^1^ (76)	↑ endogenous GH release → ↑ IGF-1; clinically ↓ VAT and may ↓ liver fat (72, 123).	Beyond approved use (HIV-associated lipodystrophy): reduction VAT and may modestly decrease hepatic fat or NAFLD; suggested preserved insulin sensitivity, modest lipid improvements, and increased GH/IGF-1 activity (72–76).	Generally well tolerated; Injection-site reactions (pain, redness); musculoskeletal symptoms (arthralgia, myalgia); elevated IGF−1 (theoretical cancer risk); glucose intolerance / insulin resistance (monitoring recommended); rare allergic reactions / peripheral edema (73, 75, 124, 125) No long term safety data available.	A	RCT evidence and FDA-approved indication (HIV-associated lipodystrophy); off-label uses unproven (71–76).	
GH fragment (C-terminal)							
AOD9604 (hGH 176–191)	p.o; short peptide; t½ not reported in human trials (84)	Designed to mimic GH lipolytic domain → minimal systemic GH/IGF−1 activation observed in humans; transient, modest metabolic changes (weight/fat) reported in some studies; no meaningful IGF−1 elevation detected (84).	Physiologic lipolytic signaling without activation of full GH/IGF−1 axis; explored for effects on body composition and weight management in humans (reducing adiposity) (84)	Generally well tolerated; mild/transient headache, fatigue, injection reactions; no significant alterations in glucose tolerance or IGF−1 observed; theoretical concerns regarding efficacy and long-term metabolic effects remain due to limited human trial data (84). No long term safety data available. ​​No established oncologic signal in clinical studies (mechanism does not significantly elevate systemic IGF-1).	B/C	Small short-term human trials with limited body-composition signals; predominantly preclinical mechanistic data (83–86).
Peptide GHS						
GHRP-6	i.v./s.c/p.o/s.l (sublingual)/i.n (intranasal) (126–128).; short-acting t½ ~ 2,5 h^2^ (129)	↑ endogenous GH release in a dose−dependent manner; peak GH responses observed within 30–75 min after oral or parenteral administration and enhanced GH secretion in combination with GHRH (127, 130, 131).	Investigational use for GH axis modulation; neuroendocrine and sleep-modulating effects; potential cytoprotective effects (preclinical data); safe short-term stimulation of GH, ACTH, and cortisol in healthy adults (91, 132)	Stimulation of pituitary-adrenal axis (ACTH/cortisol elevation); altered nocturnal hormone and sleep patterns; minor transient adverse events in short−term human testing; variable GH responses in neuroendocrine dysfunction; no long term safety data available (91, 131, 132). Mitogenic GH/IGF-1 signalling (theoretical oncologic concern); no established clinical carcinogenic signal (117).	B	Phase I and small mechanistic human studies (acute GH/PRL/ACTH responses); no controlled efficacy trials (91, 92, 126–132).
GHRP-2 (Pralmorelin)	s.c/i.v/i.n (97, 133, 134); short-acting; t½~0,55+/-0,14 h, GH peak ~60 min post-dose (135)	↑ endogenous GH secretion and ↑ appetite when administered peripherally in healthy humans (96, 97).	Beyond diagnostic use in Japan for GH deficiency (99): may be explored for ameliorating catabolic states and appetite stimulation, as evidenced by increased food intake in lean and obese subjects and reports of enhanced appetite with chronic administration in GH−deficient pediatric populations (98, 119)	In short−term clinical studies, generally well tolerated with increases in appetite reported; potential safety considerations include transient appetite, prolactin levels and no significant weight changes; no long term safety data available (90, 96–98). Mitogenic GH/IGF-1 signalling (theoretical oncologic concern); no established clinical carcinogenic signal (117).	B	Diagnostic clinical use (GHD testing); small human studies for GH/appetite endpoints (90, 96–99, 133–135).
Hexarelin	s.c./p.o/i.v/i.n (136, 137).; short-acting t½ ~55 min (138)	↑ dose-dependent and sustained GH secretion; PRL/ACTH/cortisol increase reported (90, 101)	Therapeutic potential in pediatric growth disorders, obesity−related GH deficiency, and pituitary dysfunction, with evidence of increased IGF−1 and anabolic effects in children and GH−deficient patients, suggesting possible utility in broader endocrine and growth−related conditions (102, 139).	Generally well tolerated with no serious adverse events reported; Alteration of sleep architecture (reduced slow−wave sleep); stimulation of multiple endocrine axes (ACTH, cortisol, prolactin) beyond GH; non−specific pituitary/adrenal activation observed (90, 138, 140). Theoretical mitogenic implications via IGF-1; no direct evidence of carcinogenesis in clinical studies (117). No long term safety data available.	B	Phase I/II studies in paediatric GHD and adult endocrine populations; no body-composition trials (90, 101, 102, 136–140).
Ipamorelin	i.v./s.c (104).; short-acting t½ ~ 2h (104)	↑ GH secretion in dose−dependent fashion (104)	Beyond limited clinical development in postoperative ileus, the relatively favourable tolerability profile in humans provides rationale for investigating ipamorelin in broader GH−related metabolic or catabolic conditions, although evidence in non−approved indications remains minimal (105).	Generally favourable tolerability with no significant common adverse effects reported compared to GH therapy; no long term safety data available (93, 105). Theoretical mitogenic implications via IGF-1; no direct evidence of carcinogenesis in clinical studies (117).	B	Phase I human studies and postoperative-ileus clinical development; no efficacy data in GH-related metabolic indications (103–105).
IGF-1 analogues							
PEG-MGF (pegylated mechano growth factor; IGF-1Ec-related)	No peer-reviewed clinical studies in humans have reported the route of administration; pegylation extends stability, t½ not reported (106)	IGF-1R tyrosine kinase signaling; satellite cell proliferation; preclinically described local role after mechanical stress/damage (107, 108, 110)	No peer-reviewed human studies.	No peer-reviewed human studies. Theoretical proliferative concern based on IGF-1Ec (MGF) expression in cancer biology (preclinical evidence) (141).	D	No peer-reviewed human studies.	
IGF1-LR3	No peer-reviewed clinical studies in humans have reported the route of administration; t½ not documented	Potent IGF-1 receptor activation; anabolic and mitogenic signaling; enhanced protein synthesis; stimulation of cell proliferation and survival (as its main component IGF-1) (113, 114)	No peer-reviewed human studies.	No peer-reviewed human studies. Theoretical mitogenic potential based on IGF-1/IGF-1R signalling biology (142).	D	No peer-reviewed human studies.	

^1^ after 2 weeks of daily 2-mg dosing.

^2^ The distribution phase is approximately 7,6 +/- 1,9 min, and the elimination phase is 2,5 +/- 1,1 h, resulting in an effective half-life of around 2.5 h.

*Evidence base coding (per Section 3.5 stratification): **A**, randomized controlled trial evidence within an approved clinical indication; A*, historical regulatory approval (FDA approval for paediatric GHD historically held; commercially withdrawn 2008), with no current regulatory approval for performance/body-composition indications; **B**, phase I/II human studies (PK/PD, endocrine, or paediatric GHD-related), without controlled efficacy data for body-composition or performance endpoints; **C**, limited human data, predominantly short-term or indirect; **D**, no peer-reviewed human studies, with claims resting on preclinical extrapolation and grey-literature user narratives.

**Figure 1:**
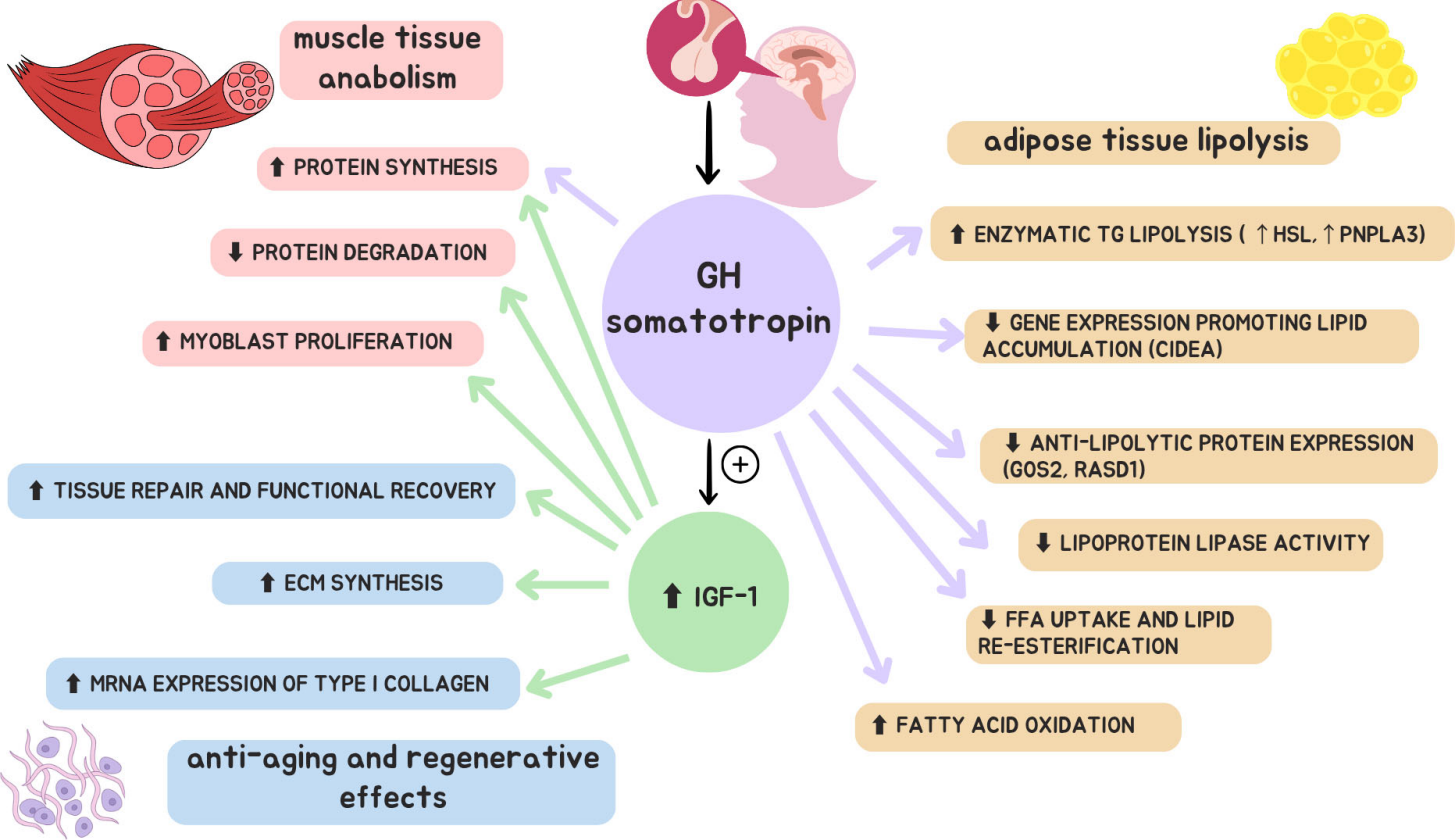
Effects of the GH-IGF-1 axis on skeletal muscle tissue, adipose tissue, and regenerative processes. Upward/downward arrows indicate relative increases/decreases. Lavender arrows denote predominantly direct GH actions; green arrows denote predominantly IGF-1–mediated actions (as depicted). The scheme is simplified and highlights selected mechanisms; effects are context-dependent (e.g., tissue, nutritional state, age, hormonal milieu). GH, growth hormone; IGF-1, insulin-like growth factor-1; TG, triacylglycerol; HSL, hormone-sensitive lipase; PNPLA3, patatin-like phospholipase domain-containing 3; CIDEA, cell death-inducing DFFA-like effector A; G0S2, G0/G1 switch gene 2; RASD1, Ras-related dexamethasone-induced 1; FFA, free fatty acids; ECM, extracellular matrix; mRNA, messenger RNA.

**Figure 2:**
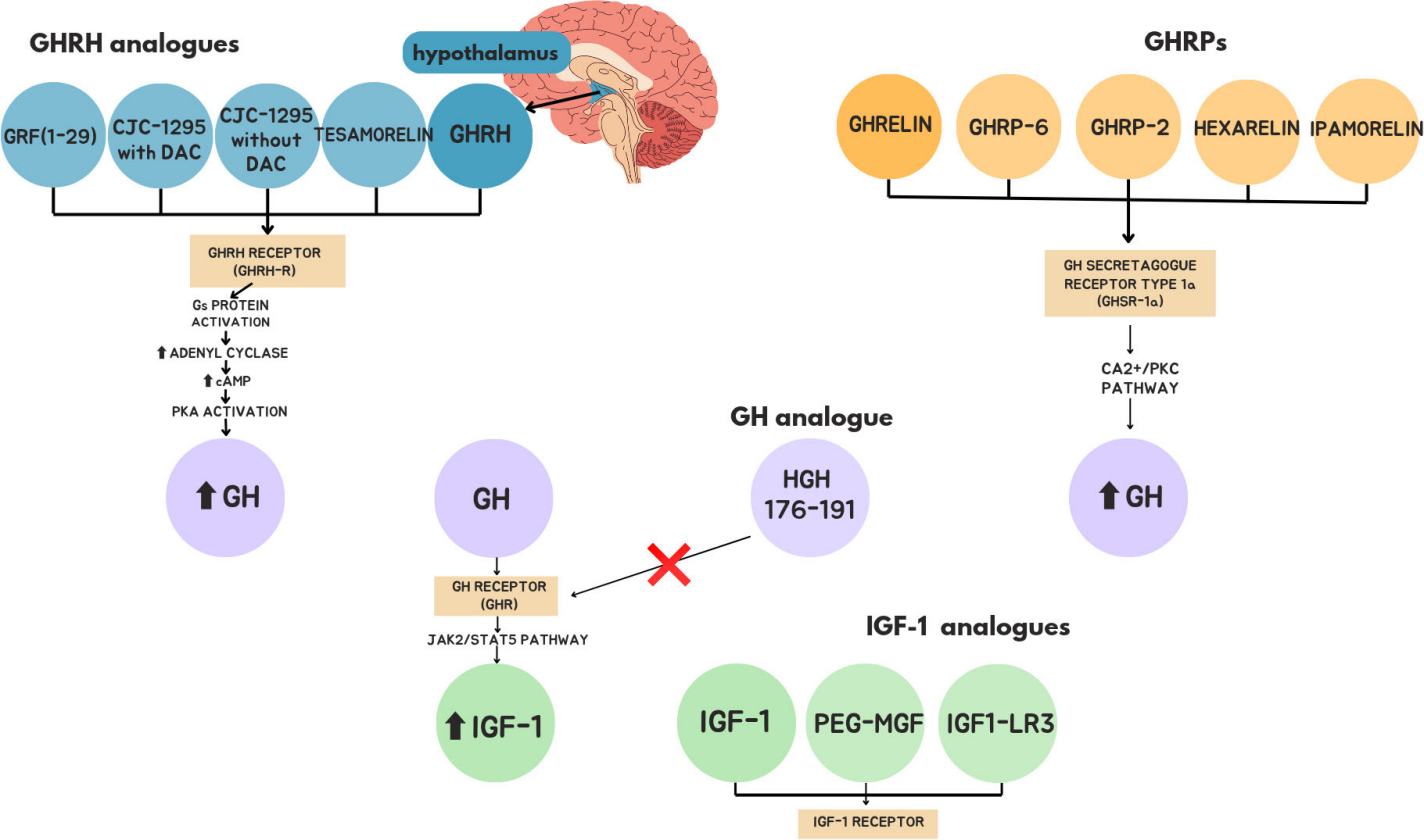
Overview of the discussed compounds and their receptors. The diagram summarises the principal receptor targets and canonical intracellular signaling pathways. Upward arrows indicate stimulation of GH secretion or increased IGF-1 signaling as depicted. The scheme is simplified; some compounds may have additional actions and context-dependent effects. GHRH, growth hormone–releasing hormone; GHRH-R, GHRH receptor; GHRP, growth hormone–releasing peptide; GHSR-1a, growth hormone secretagogue receptor type 1a; GH, growth hormone; GHR, GH receptor; IGF-1, insulin-like growth factor 1; JAK2/STAT5, Janus kinase 2/signal transducer and activator of transcription 5; cAMP, cyclic adenosine monophosphate; PKA, protein kinase A; PKC, protein kinase C; Ca²^+^, calcium; PEG-MGF, PEGylated mechano-growth factor; IGF-1R, IGF-1 receptor; DAC, drug affinity complex.

**Figure 1** illustrates a schematic representation of key downstream effects within the GH–IGF-1 signalling axis across relevant tissues. **Figure 2** provides a consolidated overview of the compounds addressed in this work and their primary receptor targets.

#### 3.4.1 GHRH analogues

Growth hormone–releasing hormone (GHRH) analogues are synthetic peptides designed to stimulate endogenous GH secretion by activating the pituitary GHRH receptor, thereby increasing GH pulsatility and secondarily IGF-1. Because they act upstream of GH release, their effects remain largely shaped by physiological regulatory loops (e.g., somatostatin tone and IGF-1–mediated negative feedback), although long-acting constructs can produce more sustained stimulation. The compounds discussed here include sermorelin, tesamorelin, and CJC-1295.

##### 3.4.1.1 Sermorelin (GRF 1-29)

Sermorelin is a GHRH analogue that has been used in the diagnostic evaluation and treatment of children with idiopathic GH deficiency, including stimulation testing of pituitary secretory capacity (66). In the United States, sermorelin acetate was previously FDA-approved (Food and Drug Administration) and marketed as *Geref*, but the product was later discontinued by the manufacturer and the corresponding approvals were withdrawn effective June 18, 2009. Importantly, FDA subsequently determined that *Geref* was not withdrawn for reasons of safety or effectiveness, which supports a non-clinical explanation for discontinuation (e.g., commercial/availability decisions) rather than a new safety signal (67, 68). In off-label use, sermorelin is commonly framed as a “physiologic” way to increase endogenous GH pulsatility for body-composition goals. However, clinically meaningful benefits in healthy individuals remain uncertain because the human evidence base is limited and largely indirect. Evidence suggesting potential gonadal downstream effects is similarly indirect: in GH- and PRL-deficient *Snell dwarf* mice, short-term administration of exogenous GH or IGF-I increased testicular LH/hCG receptor abundance and enhanced hCG-stimulated steroidogenesis without altering circulating LH or basal testosterone. The relevance to human hypogonadism remains uncertain, and such animal findings should not be extrapolated to routine treatment (69). Finally, real-world use is complicated by quality-control uncertainty and compositional variability characteristic of unregulated peptide supply chains (70).

##### 3.4.1.2 Tesamorelin

Tesamorelin is the most comprehensively characterised GHRH analogue from a regulatory and clinical-trial perspective, supported by an FDA-approved indication and a comparatively substantial randomised clinical trial evidence base in HIV-associated lipodystrophy. It was first approved in the United States in November 2010 (*Egrifta*) for the reduction of excess abdominal fat in adults with HIV and lipodystrophy, and newer presentations were later introduced to simplify preparation and administration. The most recent formulation, *Egrifta WR*, was approved in March 2025 and is positioned as a more convenient formulation (e.g., weekly reconstitution for daily administration, lower administration volume) while relying on the established tesamorelin efficacy/safety platform (71).

In HIV-infected populations with abdominal fat accumulation, clinical trials consistently demonstrate reductions in VAT, and imaging-based studies also report reductions in hepatic fat and stop the progression of fibrosis (72–74). These findings provide biological plausibility for exploring tesamorelin in broader metabolic contexts. Though evidence outside HIV cohorts remains limited, a short-term randomised, placebo-controlled trial in patients with type 2 diabetes (12 weeks; n=53) found no significant deterioration in insulin dynamics or glycaemic control with tesamorelin, while the 2 mg group showed modest reductions in total and non-HDL cholesterol (both by ~0.3 mmol/L versus placebo) (75). Moreover, a short mechanistic study in healthy men (2 weeks of tesamorelin 2 mg SC daily; n=13) demonstrated increased endogenous GH pulsatility and higher IGF-1 concentrations without a significant change in fasting glucose or insulin-stimulated glucose uptake assessed by euglycaemic clamp, suggesting preserved peripheral insulin sensitivity over short exposure (76).

In off-label fitness discourse, tesamorelin is frequently positioned as a tool for improving body composition and “recovery”, and sometimes for anti-inflammatory or tissue-healing goals. These claims, however, extend beyond the endpoints of the RCT trials and should be presented cautiously as marketing narratives rather than established clinical effects. Notably, tesamorelin is being investigated as an adjunct to enhance recovery after upper-extremity peripheral nerve injury repair in an ongoing phase 2 randomised study (NCT03150511; recruiting; planned n=36), evaluating functional motor and sensory outcomes over 12 months; results are not yet available (77).

Importantly, tesamorelin’s safety profile was assessed during RCTs. Across studies, treatment is generally well tolerated, with predominantly mild-to-moderate adverse effects (75, 78, 79). Importantly, while tesamorelin was generally well tolerated in randomized clinical trials, key endocrine and metabolic precautions - including IGF-1 elevation thresholds, fluid retention syndromes, and warnings regarding glucose intolerance - are derived primarily from regulatory prescribing information and pharmacokinetic–pharmacodynamic evaluations rather than from peer-reviewed randomized trials (71).

##### 3.4.1.3 CJC-1295 (with and without DAC)

CJC-1295 is a synthetic analogue of GHRH, designed to stimulate pituitary GH secretion and secondarily increase circulating IGF-1. Compared with native human GHRH, CJC-1295 includes four amino-acid substitutions that increase resistance to proteolytic degradation (80).

Two main variants circulate commercially in the off-label market: CJC-1295 with a drug-affinity complex (DAC) and CJC-1295 without DAC. The DAC modification enables binding to circulating albumin, markedly prolonging exposure and enabling a more sustained pharmacodynamic effect after a single administration. This prolonged stimulation results in longer-lasting elevations of GH and IGF-1 in healthy adults.

The best human evidence comes from randomised, placebo-controlled, double-blind ascending-dose trials conducted in healthy adults using CJC-1295 DAC administered subcutaneously. In these studies, a single injection produced dose-dependent increases in mean plasma GH (approximately 2- to 10-fold for ≥6 days) and mean plasma IGF-1 (approximately 1.5- to 3-fold for 9–11 days). No serious adverse reactions were reported, and tolerability was described as generally favourable in the tested dose range (80).

By contrast, “CJC-1295 without DAC” remains essentially uncharacterised in the peer-reviewed human literature. To date, no controlled clinical studies have directly evaluated this compound in humans, and assertions regarding physiologic GH pulsatility or improvements in body composition derive largely from extrapolation from related compounds (primarily GHRH(1–29), known as sermorelin, as previously described), as well as from non-academic sources, rather than from direct clinical evidence (81). Consequently, there is currently no peer-reviewed support for clinically meaningful effects on body composition or performance outcomes.

Importantly, neither CJC-1295 variant is approved as a therapeutic agent by major regulatory authorities (e.g., FDA, EMA). Moreover, FDA listed it as prohibited on “Category 2 of Bulk Drug Substances List” due to vasodilatatory reactions and tachycardia reports (82).

#### 3.4.2 GH-derived fragment: AOD9604/hGH(177–191)/hGH(176–191)/GH fragment

AOD9604 is a synthetic C-terminal fragment of human GH, often described as hGH(177–191) with an additional N-terminal tyrosine, hence sometimes labelled “hGH 176–191” or just “GH fragment”. It was developed as an “anti-obesity” candidate intended to retain GH’s lipolytic/antilipogenic signalling while avoiding classical GH receptor–mediated growth effects and IGF-1–driven systemic exposure. In preclinical models, chronic subcutaneous administration of AOD9604 increased fat oxidation and markers of lipolysis and attenuated weight gain in obese mice. Importantly, unlike full-length GH, it did not induce hyperglycaemia and showed no GH receptor binding or GH receptor–mediated proliferative activity *in vitro (*83*).*

Human evidence is limited and largely short-term. As an oral anti-obesity drug candidate, in a 24-week study (n=534, 502 randomized) it failed to demonstrate a significant benefit on the primary weight-loss endpoint (84, 85). Importantly, conclusions regarding the lack of clinical efficacy in obesity are based primarily on sponsor-reported phase 2 programme outcomes rather than peer-reviewed randomized efficacy trials (85). Consequently, there are no robust data supporting clinically meaningful visceral-fat reduction or broader metabolic benefit in humans, and extrapolations to “recomposition” or performance outcomes remain speculative.

Claims around osteoarthritis, “joint repair,” or tissue regeneration are currently grounded mainly in animal work (e.g., intra-articular AOD9604 ± hyaluronic acid improving cartilage-related outcomes in a rabbit OA model), with a lack of convincing human clinical trial evidence in these indications (86). AOD9604 is not approved by major regulatory agencies for any indication and similar to CJC-1295 is on the “Category 2 of Bulk Drug Substances List” due to immunogenicity (82).

#### 3.4.3 GHRP - peptide GH secretagogues

Growth hormone–releasing peptides (GHRPs) are short synthetic peptides that stimulate endogenous GH secretion by activating the ghrelin receptor - the growth hormone secretagogue receptor type 1a (GHSR-1a), at the level of the hypothalamus and anterior pituitary. Beyond the central GH axis, GHSR-1a is also expressed in multiple peripheral tissues (including pancreatic islets, thyroid, adrenal tissue, adipose tissue, and myocardium), which provides biological plausibility for pleiotropic effects beyond GH release. A second molecular target described for certain GHRPs is CD36, a scavenger receptor expressed in endothelial and immune cells, adipocytes, hepatocytes, and skeletal/cardiac muscle (87, 88). Experimental studies indicate that GHRPs can enhance GH secretion while largely preserving physiological regulatory loops within the GH–IGF-1 axis, including somatostatin tone and IGF-1–mediated negative feedback, in contrast to exogenous recombinant GH administration (89). The GHRP class discussed in this review includes GHRP-6, GHRP-2, hexarelin, and ipamorelin.

##### 3.4.3.1 GHRP-6

GHRP-6 is a synthetic hexapeptide and one of the earliest growth hormone–releasing peptides evaluated in humans, serving as a prototype for the development of later analogues. In controlled human studies, GHRP-6 robustly stimulates pulsatile GH secretion, consistent with its role as a ghrelin receptor (GHSR-1a) agonist (90–92). Off-label narratives often extrapolate from this endocrine response to claims of improved “recomposition” through enhanced protein synthesis and lipolysis. However, direct evidence for meaningful changes in body composition outcomes in humans remains limited and should be interpreted cautiously. A characteristic and clinically relevant effect of GHRP-6 is a consistent appetite-stimulating response, reflecting strong orexigenic signalling through the ghrelin pathway (87, 93). In addition, transient increases in cortisol and prolactin have been reported, which may be relevant when considering potential endocrine side effects or confounding of hormonal assessments (90, 91). Experimental work also suggests that GHRP-6 may influence sleep architecture, with reported effects on Rapid Eye Movement (REM)/Non-Rapid Eye Movement (NREM) patterns, supporting central neuroendocrine activity beyond GH release (91). Preclinical literature describes potential cardioprotective actions - such as reduced infarct size and protection of cardiomyocytes under ischaemic conditions, but these findings derive from animal and *in vitro* models and currently lack confirmatory evidence from human clinical trials (94, 95).

##### 3.4.3.2 GHRP-2

GHRP-2 (pralmorelin) is a GHSR-1a agonist that stimulates endogenous GH secretion. GHRP-2 has been shown to increase food intake under experimental conditions, indicating an orexigenic effect mediated via ghrelin signalling, an acute response that has been documented both in obese subjects and in healthy men and is comparable in magnitude to that induced by ghrelin itself (96, 97). Similar to other GHRPs, administration of GHRP-2 has been associated with transient increases in cortisol and prolactin, which may be clinically relevant when interpreting endocrine testing or considering potential adverse endocrine effects (90). Longer-term oral administration in children with growth hormone deficiency has been associated with increased appetite and modest weight gain, underscoring potential effects on energy balance in clinical populations (98). A key distinction from many peptides that remain confined to experimental or non-clinical use is that pralmorelin has an established clinical diagnostic role in Japan, as a pharmacologic stimulus to assess growth hormone deficiency and hypothalamic–pituitary function (99, 100). Beyond its established diagnostic application, broader therapeutic use of GHRP-2 remains limited and is not supported by widespread regulatory approval.

##### 3.4.3.3 Hexarelin

Hexarelin is a synthetic growth hormone–releasing peptide structurally related to GHRP-6, characterised by chemical modifications that confer high biological activity. In human studies, hexarelin potently stimulates GH secretion and, similarly to other GH-releasing peptides, also activates prolactin release as well as the corticotropic axis, leading to increases in ACTH and cortisol levels (90, 101). These observations highlight that, despite its efficacy as a GH secretagogue, hexarelin induces broader endocrine responses, underscoring the relevance of hormonal cross-talk associated with this class of compounds (90, 101). Evidence for downstream anabolic or body-composition–related effects of hexarelin in humans is limited and largely derived from its capacity to increase GH and IGF-1, rather than from controlled trials specifically designed to assess body composition or physical performance outcomes. In a paediatric population with growth disorders, several months of intranasal hexarelin administration were associated with increased IGF-1 concentrations, weight gain, and changes in anthropometric and skinfold measurements, including reductions in skinfold thickness (102). While these findings may suggest potential anabolic or body-composition–related effects, they originate from a specific clinical population and do not establish a generalisable or approved therapeutic protocol, particularly for healthy individuals (102).

##### 3.4.3.4 Ipamorelin

Ipamorelin is a synthetic GH secretagogue that acts as a relatively selective agonist of the GHSR-1a receptor, stimulating endogenous GH release while producing comparatively minimal activation of other pituitary–adrenal axes in available human studies (103). In a phase I study in healthy adults, ipamorelin was generally well tolerated and produced a clear GH response, with peak GH levels occurring approximately 40–60 minutes after administration; importantly, no significant effects on other pituitary or adrenal hormones were reported in that setting (104). This relatively “cleaner” endocrine profile is often presented as a practical distinction from earlier secretagogues with broader hormonal effects.

Although ipamorelin demonstrated prokinetic effects in preclinical models, results from a randomized, controlled proof-of-concept clinical study in patients undergoing bowel resection did not show a statistically significant improvement in postoperative gastrointestinal recovery compared with placebo. These findings suggested limited clinical efficacy in this setting and tempered further clinical investigation of ipamorelin for the management of postoperative ileus (105).

In off-label fitness discourse, ipamorelin is also frequently discussed in combination with CJC-1295 (typically the non-DAC form) to amplify GH release through complementary stimulation of the GH axis. However, this practice is largely driven by anecdotal rationale, and controlled clinical evidence supporting synergy or meaningful body-composition outcomes in healthy individuals remains limited (93).

#### 3.4.4 IGF-1 analogues

The final group discussed in this review comprises IGF-1 analogues, represented here by pegylated mechano growth factor (PEG-MGF) and IGF-1 LR3 (insulin-like growth factor-1, long [Arg3]). These compounds act primarily through the IGF-1R, a transmembrane receptor with intrinsic tyrosine kinase activity that mediates many of the downstream “somatomedin” effects of IGF-1, as outlined earlier in this manuscript. Consequently, IGF-1 analogues are expected to reproduce core IGF-1 biological actions (to varying degrees depending on formulation, bioavailability, and exposure profile), and their potential benefits and risks should be interpreted in the context of systemic IGF-1 signalling rather than GH physiology alone.

##### 3.4.4.1 PEG-MGF

PEG-MGF is marketed as a PEGylated form of mechano-growth factor (MGF). In physiological terms, MGF corresponds to an IGF-1 splice variant (often referred to as IGF-1Ec) discussed as part of the broader, tissue-specific IGF-1 isoform system (106). The rationale behind PEGylation (as promoted commercially) is to increase molecular stability and prolong exposure, however the mechanistic literature most often cited in support of “MGF-like” regenerative effects is largely based on IGF-1Ec/MGF biology and synthetic E-domain peptide constructs, rather than on PEG-MGF as a clinically characterised drug entity (106). Importantly, PEGylation fundamentally alters pharmacokinetics and tissue exposure, such that biological effects observed with native IGF-1Ec or isolated E-domain peptides cannot be assumed to apply to PEG-conjugated constructs.

In skeletal muscle, experimental work indicates that the IGF-1Ec (MGF) peptide and mature IGF-1 may exert non-identical effects on myoblast biology, with differential roles in proliferation and differentiation - an observation frequently used to justify the concept of “repair-biased” IGF signalling (107). Consistent with this line of reasoning, a synthetic MGF E-peptide has been reported to enhance outcomes in myogenic precursor cell transplantation models, supporting biological plausibility for a role in muscle regeneration pathways under experimental conditions (108).

Cardiac preclinical studies extend this “repair signal” narrative to the myocardium. Localised delivery of an MGF E-domain peptide has been shown to improve cardiac function following myocardial infarction in animal models (109), and related work suggests that the E-domain region may inhibit apoptosis and help preserve cardiac function during ischaemic injury (110). Together, these data support the idea that MGF/E-domain–derived peptides can influence tissue repair programmes in controlled experimental settings.

Crucially for clinical interpretation, the current evidence base is predominantly preclinical and heterogenous (isoform biology vs synthetic peptide fragments vs engineered formulations), and the field includes unresolved questions about mechanisms, translatability, and safety - points highlighted in review-level discussions of MGF as a potential “repair” signal (106). As such, claims that PEG-MGF reliably accelerates recovery or improves body composition in humans remain unproven, and robust RCTs in human populations are lacking. Reports on internet forums describing “local-only action” and an absence of systemic adverse effects should be treated as anecdotal and cannot substitute for controlled safety or efficacy data.

##### 3.4.4.2 IGF-1 LR3

IGF-1 LR3 is an engineered analogue of IGF-1 characterised by the substitution of glutamic acid with arginine at position 3 and the presence of an additional N-terminal amino acid extension. These structural modifications result in a markedly reduced affinity for IGF-1-binding proteins compared with native IGF-1, as demonstrated in studies using biological model systems (111, 112). At the mechanistic level, IGF-1 acts primarily as an agonist at IGF-1R, activating canonical anabolic and mitogenic pathways including PI3K–Akt and MAPK/ERK, which influence protein synthesis, cell-cycle regulation, and cell survival. As IGF-1 LR3 engages the same receptor, it is generally presumed to elicit comparable downstream signalling, although its altered binding characteristics may influence the magnitude or temporal dynamics of pathway activation. These pathways are well characterised in cell biology and oncology models and explain why IGF-1 signalling is often framed as “anabolic,” but they also underpin theoretical concerns about proliferative signalling in susceptible tissues (113, 114). Despite strong biological plausibility, direct interventional evidence in healthy humans demonstrating consistent improvements in skeletal muscle mass or strength with exogenous IGF-1 is limited. Trials performed in clinical populations (e.g., neuromuscular disorders) provide a useful reality check: interventions combining IGF-1 with IGFBP-3 have been associated with increases in lean body mass without clear improvements in functional outcomes. This underscores that anabolic signalling and body recomposition do not necessarily translate into clinically meaningful physical performance gains (115). *In vitro* data in human muscle cells show that IGF-1 can increase myotube diameter and support growth-related cellular phenotypes, but these findings cannot be assumed to predict effects in whole-body human physiology (116). From a clinical safety perspective, two domains deserve emphasis. First, reported adverse effects in off-label settings frequently centre on insulin-mimetic activity (e.g., symptoms suggestive of hypoglycaemia), which is biologically plausible given receptor cross-talk within the insulin/IGF axis, however, many such reports originate from non-clinical sources and should be treated cautiously. Second, long-term cancer risk cannot be inferred directly from short-term exposure, but epidemiologic data linking higher circulating IGF-1 with increased risk of several cancers are often cited to justify concern that exogenous IGF-1 analogues could modulate tumour biology in predisposed individuals. This risk framing should be presented as theoretical but clinically relevant, especially in the absence of long-term safety data for off-label IGF-1 analogue use (117).

### 3.5 Evidence stratification across compounds

The evidence base for GH–IGF-1-axis performance-enhancing peptides is highly uneven and should not be interpreted as a unified body of human efficacy data. To make this asymmetry navigable for clinicians, every compound reviewed has been assigned to one of four evidentiary tiers, summarised in the evidence base column of **Table 1**. At one end of the spectrum, tesamorelin (tier A) is supported by randomized controlled trials within an FDA-approved clinical indication. At the opposite end, CJC-1295 without DAC, PEG-MGF, and IGF-1 LR3 (tier D) have no peer-reviewed human studies and are supported only by preclinical extrapolation and grey-literature user narratives. Most other GHRH analogues and ghrelin-mimetic secretagogues occupy an intermediate tier (B), with phase I/II human studies that do not address performance- or body-composition endpoints, while AOD9604 stands apart as a tier-B/C agent for which short-term human trials exist but with limited interpretable signals.

The methodological constraints affecting all tiers: short follow-up, small samples, non-performance endpoints, and the absence of standardized exposure verification in off-label users - are addressed in detail in the dedicated Limitations section.

### 3.6 Navigating the patient-encounter: interpreting unregulated dosing protocols

While clinical data for several compounds discussed in this review are limited to early-phase studies or indirect evidence, user-oriented platforms provide detailed “stacking,” cycling, and dosing narratives that are often presented with a level of certainty not supported by the underlying data. In clinical practice, these protocols function as a de facto decision framework for patients, yet they frequently extrapolate from preclinical findings, short-term physiologic studies, or anecdotal accounts. As a result, the endocrinologist is increasingly asked to translate an online regimen into a medically defensible risk assessment, while acknowledging two recurring uncertainties: **(I)** the *evidence gap* (limited human efficacy/safety data for physique- or performance-related aims) and **(II)** the *identity gap* (variable composition, sterility, and purity in unregulated supply chains).

A practical first step is to create conditions for disclosure and a usable exposure history. Many patients will not initially label these products as “drugs,” and some may omit co-use of other performance-enhancing agents that ultimately dominates the clinical picture. A neutral, safety-focused approach, framed around accurate interpretation of symptoms and laboratory findings, often improves the quality of history-taking. Clinically relevant exposure details include the presumed compound class (GHRH analogue vs secretagogue vs GH fragment vs IGF-1 analogue), whether agents were combined (“stacked”), the time course (initiation, escalation, cycle duration, and time since last use), route and injection practices, and potential co-exposures (particularly anabolic-androgenic steroids, thyroid hormone, stimulants, diuretics, and metabolic agents). Baseline cardiometabolic risk (obesity, sleep apnoea, prior dysglycaemia, hypertension) materially modifies the likelihood that endocrine-metabolic adverse effects will become clinically significant.

Symptom triage benefits from grouping nonspecific complaints into domains that map onto plausible endocrine and metabolic mechanisms discussed throughout the manuscript. Fluid retention syndromes (peripheral edema, paresthesias, arthralgia/myalgia; as documented in peer-reviewed human studies exclusively for tesamorelin, with comparable findings not identified in the available peer-reviewed literature for the other peptides discussed, although mechanistic overlap within the somatotropic axis warrants cautious interpretation), merit careful differentiation from cardiac, renal, hepatic, and thyroid contributors, especially when dyspnoea or reduced exercise tolerance is present. Metabolic complaints (increased appetite, polyuria/polydipsia, fatigue or “crashes,” reduced training tolerance) should prompt objective evaluation for dysglycaemia, recognizing that insulin resistance risk may be amplified by sustained stimulation of the somatotropic axis and by common co-exposures. Injection-site reactions and systemic inflammatory symptoms should be assessed with a low threshold to consider infectious complications, given that sterility cannot be assumed in informal distribution channels.

Laboratory interpretation in this context is vulnerable to common pitfalls. Because circulating GH is pulsatile and short-lived, isolated random GH measurements are often uninformative, whereas IGF-1 - while imperfect - typically provides a more interpretable integrated signal of pathway activation over time when measured and interpreted using age-adjusted reference ranges. Even IGF-1 should be interpreted cautiously in the setting of altered nutrition, hepatic dysfunction, thyroid disease, or acute illness. When prolactin or cortisol abnormalities are encountered in a patient who uses PEPs modulating the GH-IGF1 axis, careful retesting after PEP withdrawal is encouraged before escalating to extensive endocrine imaging or dynamic testing. It is important to remember that mild-to-moderate alterations in prolactin or cortisol serum concentrations can also reflect stress, sleep disruption, medication effects, and assay factors, rather than primary pituitary or adrenal pathology. From a pragmatic standpoint, an initial evaluation often prioritizes patient safety (e.g., objective glycaemic status and basic organ function) and then proceeds to targeted endocrine testing guided by symptoms, exposure class, and pre-test probability.

Counselling represents the core “bridge” between clinical evidence and real-world protocols. Patients frequently present with protocol-based causal narratives (e.g., attributing a broad symptom cluster to a single peptide), whereas the clinician’s task is to anchor on differential diagnosis, identify objective harm signals, and communicate uncertainty clearly. A useful frame is to separate (I) what is known (physiologic effects demonstrated in controlled settings for some agents), (II) what remains unproven (durable improvements in strength, hypertrophy, or recovery in healthy individuals for many compounds), (III) what is intrinsically uncertain in unregulated markets (product identity, purity, and batch variability), and (IV) what can be harmful (lack of an established safety profile or only limited short-term safety data, no long term follow-up data, potential oncological risks from long-term GH/IGF-1 axis stimulation). Importantly, the clinician can support harm identification and monitoring without implicitly endorsing use. This boundary should be explicit, particularly when patients request regimen optimization rather than risk assessment.

**Figure 3:**
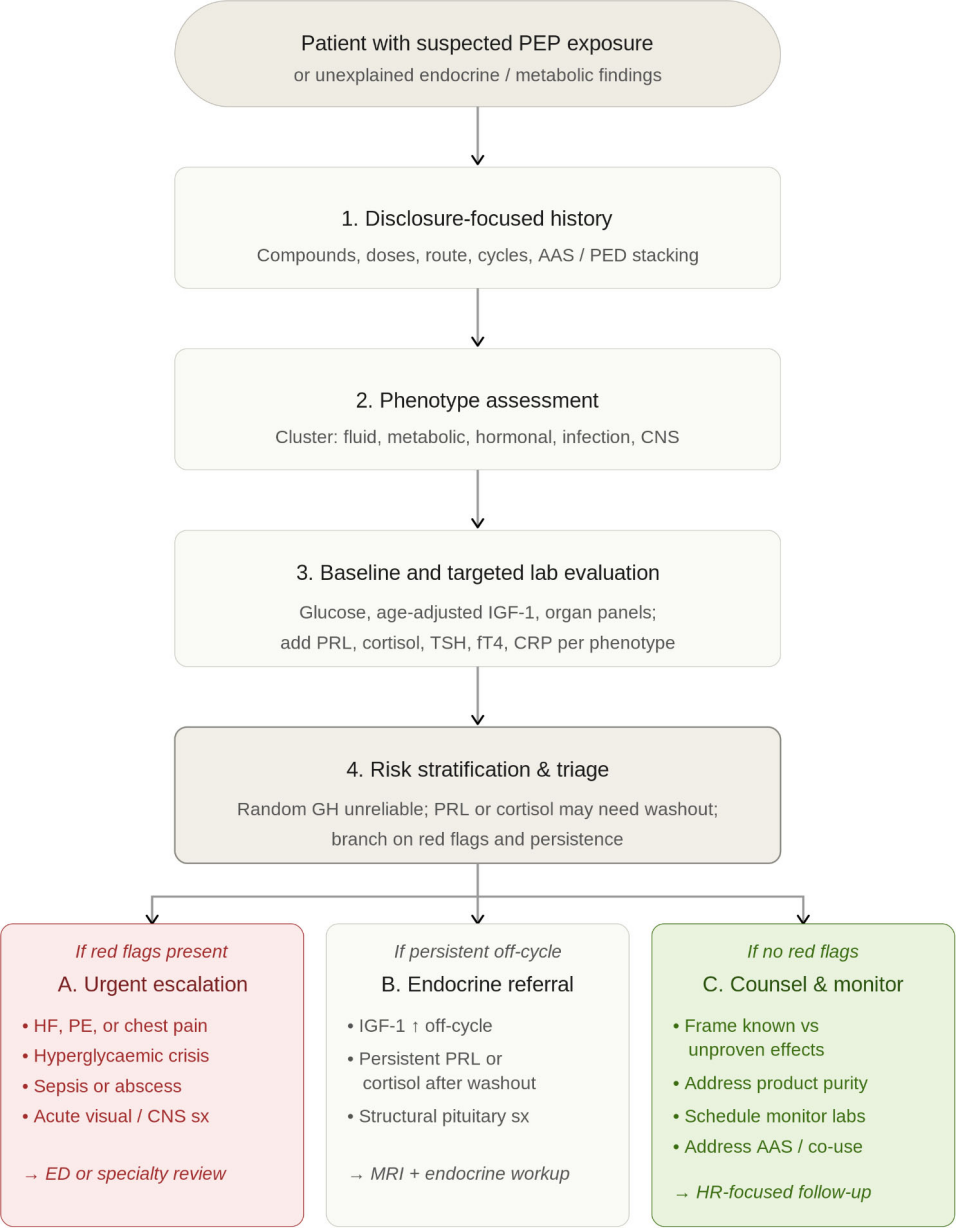
Clinical algorithm of PEPs use assessment. AAS, anabolic-androgenic steroids; CNS, central nervous system; CRP, C-reactive protein; ED, emergency department; fT4, free thyroxine; GH, growth hormone; HF, heart failure; HR, harm reduction; IGF-1, insulin-like growth factor 1; MRI, magnetic resonance imaging; PE, pulmonary embolism; PED, performance-enhancing drug; PEP, performance-enhancing peptide; PRL, prolactin; sx, symptoms; TSH, thyroid-stimulating hormone.

Finally, clinicians should maintain a low threshold for escalation when red flags emerge, including severe dyspnoea or cardiopulmonary symptoms in the setting of rapid fluid retention, marked hyperglycaemia (or symptoms suggestive of metabolic decompensation), progressive injection-site inflammation with systemic features, or neurological symptoms such as severe headaches with visual changes. **Figure 3** presents this algorithm in a visual decision-tree form, while **Supplementary Table 2** expands each step with detailed rationale. Together, they translate the endocrine and metabolic disruption patterns described in this review into a pragmatic bedside approach. The algorithm integrates peer-reviewed data on GH–IGF-1-axis agents, recognised adverse-effect profiles of anabolic-androgenic steroids, and general principles of endocrine emergency assessment rather than reproducing any single existing guideline.

**Table 2**. complements this overview by presenting dosing regimens reported in non-clinical fitness- and bodybuilding-related online sources alongside regimens used in peer-reviewed studies, illustrating the magnitude of variability, the limitations of protocol−driven self−administration outside regulated clinical contexts, and the potential for both under− and overdosing in real−world use. The online protocols included in this table are not evidence-based treatment strategies, MUST NOT be interpreted as clinically validated or safe, and are presented solely as anthropological/behavioral data describing self-administration practices and the informational environment encountered in real-world clinical care.

**Table 2:** Examples of self−reported online dosing patterns for GH–IGF−1−axis peptides (anthropological/behavioural data, not clinical recommendations).

Compound	Suggested dosage and administration*
GHRH analogues	
Sermorelin (GRF 1–29)	GH stimulation testing: 1 µg/kg IV (66).; Growth deficiency treatment: ~30 µg/kg SC (66, 143).; Bodybuilding-related forum: 200–300 µg SC, once daily, typically before bedtime on an empty stomach (144).
CJC-1295 (with DAC)	30 or 60 µg/kg SC, 2–3 times weekly or biweekly (80). Bodybuilding-related forums: 1–2 mg/week SC in 1–2 injections; typical cycles: 8–12 weeks or 12–16 weeks on, followed by 4–6 weeks off (145, 146).
CJC-1295 (without DAC)** (Mod GRF 1–29)	Bodybuilding-related forum: 100–200 µg per injection SC, 1–2 times daily, often used in combination with Ipamorelin to increase pulsatile GH release; preferably post−strength training. Typical cycles: 12–16 weeks on, followed by 4–6 weeks off (146).
Tesamorelin	1 or 2 mg SC once daily, for 2 weeks or 12 weeks or 12 months (75, 76, 147). Bodybuilding-related forum: 2 mg SC once daily; typical cycle: 8–12 weeks (148).
GH fragment (C-terminal)	
AOD9604 (hGH 176–191)	25-400 µg IV infusion in single administration (84). Obese patients: 1 mg PO once daily, for 12 weeks (149). Bodybuilding-related forums: 250–500 µg/day SC in 2–3 injections, ideally before exercise or at morning (fasted); typical cycle: 8–12 weeks for body composition (150, 151).
Peptide GHS	
GHRP-6	1 µg/kg IV bolus, single administration (126). Short-stature children: 300 µg/kg PO, single administration (127). Bodybuilding-related forum: 100–300 µg SC or IM, 2–3 times daily (fasted) to avoid insulin-mediated GH inhibition; typical cycle 8–12 weeks for body composition (152).
GHRP-2 (Pralmorelin)	Short-stature children: 1 µg/kg IV, single administration (135). Bodybuilding-related forum: 100–150 µg SC, 2–3 times daily (fasted) to avoid insulin-mediated GH inhibition; typical cycle 8–12 weeks for body composition (153).
Hexarelin	0-1 µg/kg IV bolus (dose-escalation) (101). 1,5 µg/kg SC, 2–3 times daily, for 24h (136). Bodybuilding-related forums: 100-200 µg SC 1–2 injections/day; typical cycle: 8–12 weeks on, followed by 4 weeks off (154, 155).
Ipamorelin	4.21–140.45 nmol/kg IV infusion, single administration (104). Bodybuilding-related forums: 200-300 µg SC 2 or 3 injections/day, timed to mimic natural GH pulses, often used in combination with CJC-1295 (without DAC); typical cycle: 8–12 weeks on, followed by 4–6 weeks off (156, 157).
IGF-1 analogues	
PEG-MGF**	Bodybuilding-related forums: SC or IM 200–400 µg 2–3×/week post−exercise (30–60 min after training); loading protocol: 100 µg × 2 weeks → 200 µg × 2 weeks → 300 µg × 4 weeks; maintenance: 200–400 µg/day; typical cycles 8–16 weeks with ~4−week washout (158).
IGF1-LR3**	Bodybuilding-related forums: SC or IM 20-40 µg/day, once daily, ideally post-workout; typical cycle: 4–6 weeks on, followed by an equal off-period (159).

Reported dosing regimens for various peptides are summarized from peer−reviewed studies as well as anecdotal reports from fitness− and bodybuilding−related online forums and industry sources, derived from non−peer−reviewed, often internally inconsistent online protocols Values presented include user−reported practices and experimental data and MUST NOT be interpreted as clinically validated, regulatory−approved, or safe for use. The following table is provided for descriptive and informational purposes only and does not constitute a recommendation for administration, dosing, or use of these compounds. These regimens are summarised solely to help clinicians contextualise patient−reported protocols and to recognise the scale and structure of off−label use.

*Administration refers exclusively to the route of administration as reported in the cited sources. SC denotes subcutaneous administration, IM denotes intramuscular administration, IV denotes intravenous administration, whereas PO denotes oral administration. No other routes of administration are considered or implied.

**No peer−reviewed clinical studies currently report specific dosing regimens in humans.

### 3.7 Common endocrine disruption patterns across PEP classes

Across PEP classes, endocrine and metabolic disturbances cluster into a limited set of recurring patterns rather than into compound-unique syndromes. Recognising these patterns matters clinically because patients frequently present with symptoms or laboratory abnormalities while the exact compound, dose, or stack is uncertain. **Table 3** maps, in qualitative terms, how each class of GH–IGF-1-axis PEPs relates to six recurring endocrine signals, and contrasts the within-class variability that often determines clinical relevance. Per-compound evidence and primary references are provided in sections 3.4.1–3.4.4 and **Table 1**. The descriptors below reflect the strength and consistency of available human data rather than a formal grading scheme.

**Table 3:** Cross-class endocrine and metabolic signals across GH–IGF-1-axis PEPs.

Endocrine signal	GHRH analogues	GHRPs	IGF-1 analogues	Clinical signature
GH / IGF-1 overactivation	↑↑ documented tesamorelin, CJC-1295/DAC	↑↑ documented acute pulses, phase I	theoretical no human data for PEG-MGF, IGF-1 LR3	Unifying class signal; chronic IGF-1 elevation drives the central long-term concern.
Dysglycaemia / insulin resistance	↑ variable tesamorelin: no major signal; GH-excess analogy	theoretical orexigenic-mediated weight gain	theoretical hypoglycaemia possible	Fasting glucose or HbA1c worth obtaining regardless of compound class.
ACTH / cortisol activation	sparing no consistent signal	↑↑ documented GHRP-2 / -6, hexarelin; ipamorelin sparing	theoretical not characterised	Mild hypercortisolemia → consider GHRP class effect; retest after washout before pursuing imaging.
Prolactin elevation	sparing	↑ variable GHRP-6 / -2 / hexarelin; ipamorelin sparing	theoretical	Mild hyperprolactinaemia in a PEP user → repeat off-cycle before pituitary imaging.
Fluid retention / soft-tissue	↑ documented tesamorelin; GH analogy	theoretical via GH-axis activation	theoretical via GH-axis crosstalk	Edema, paresthesia, arthralgia → first exclude cardiac, renal, hepatic, thyroid drivers.
Appetite / body composition	↑ variable limited direct effect	↑↑ documented orexigenic GHRP-2 / -6; ipamorelin less	theoretical	Rapid appetite or weight change in a PEP user → consider ghrelin-mimetic GHRP exposure.

Descriptors reflect the strength and consistency of available human data, not a formal grading scheme. ↑↑ documented = consistent signal in phase I or RCT data; ↑ variable = signal present but inconsistent between studies or agents; sparing = absence of effect documented; theoretical = inferred from mechanism or extrapolation, no direct human evidence. AOD9604 (GH 176–191 fragment) is omitted as a separate column because it acts independently of the GH receptor and shows no systematic somatotropic activation in available data; see section 3.4.2. Per-compound evidence and references: see sections 3.4.1–3.4.4 and **Table 1**.

Three implications follow for bedside reasoning. First, somatotropic activation - increased GH pulsatility, elevated IGF-1, and downstream mitogenic and metabolic signalling, is the unifying class effect, even when the specific peptide is unknown. Second, ACTH/cortisol and prolactin elevations are largely a feature of earlier ghrelin-mimetic GHRPs (GHRP-2, GHRP-6, hexarelin), with ipamorelin notably sparing - a distinction worth recovering from the exposure history before pursuing extensive pituitary imaging in mildly abnormal labs. Third, claims about IGF-1 analogues (PEG-MGF, IGF-1 LR3) and CJC-1295 without DAC rest on extrapolation rather than direct human data; clinicians should treat exposure to these compounds as evidence-poor and frame counselling accordingly. **Figure 3** translates these patterns into a stepwise bedside approach with red-flag triggers for urgent escalation.

### 3.8 Co-use with anabolic-androgenic steroids and other agents

Although this review focuses on GH–IGF−1−axis peptides, their use in practice commonly occurs within broader polypharmacy patterns that include anabolic−androgenic steroids, thyroid hormone, stimulants, insulin−sensitizing agents, diuretics, and recreational drugs (1–12). This matters because the clinical presentation attributed by patients to a single peptide may in fact reflect additive, synergistic, or confounded effects from multiple co−exposures (4–12).

The combination with anabolic−androgenic steroids deserves particular attention. AAS co−use is common in physique−oriented settings and introduces overlapping risks involving dyslipidaemia, erythrocytosis, blood pressure elevation, fertility suppression, psychiatric effects, and prothrombotic changes. Concurrent GH/IGF−1−axis manipulation may further exacerbate edema, glucose dysregulation, soft−tissue symptoms, and body−composition changes.

Clinical symptoms reported by patients using performance-enhancing substances should not be presumptively attributed to a single peptide but interpreted within the broader context of potential polypharmacy. Clinically, exposure history should therefore include explicit questioning about AAS, thyroid hormones, stimulants, and adjunctive metabolic agents rather than assuming peptide monotherapy.

### 3.9 Oncologic risk framing

Oncologic risk warrants dedicated and thorough consideration, as chronic stimulation of the GH–IGF-1 axis represents one of the most clinically significant theoretical concerns in the PEP field. IGF−1 receptor signaling activates proliferative and anti−apoptotic pathways, including PI3K/Akt and MAPK/ERK, and contributes to cell−cycle progression, survival, and invasion (160–163). Epidemiologic analyses link higher circulating IGF−1 concentrations with increased risk of several cancers, including breast and prostate cancer, and with altered overall cancer risk and mortality (117, 164, 165).

This does not establish that any specific PEP causes cancer in humans. However, in the absence of high-quality randomized controlled trials and robust long-term safety data, oncologic risk remains a legitimate concern. In the setting of unapproved long−term use, uncertain dose exposure, and frequent stacking, repeated or sustained elevation of IGF−1 should be considered as a biologically plausible risk amplifier, particularly in individuals with prior malignancy, strong family history, premalignant conditions, or other reasons for increased baseline cancer susceptibility (160, 166).

Given the absence of regulatory approval, the lack of dose standardization, and the limited availability of long-term safety data, patients should be counseled by medical professionals regarding the uncertain oncologic risks associated with sustained activation of the GH–IGF-1 signaling pathway. Discontinuation or avoidance of such compounds should generally be recommended. Current evidence does not support routine use of intensive oncologic screening solely on the basis of elevated IGF-1 or exposure to GH–IGF-1–stimulating peptides in otherwise asymptomatic individuals. Instead, clinicians should review patient’s cancer history and potential risk-factors, counsel about the uncertainty of long-term exposure, and discuss potential oncological risks. A low threshold for oncologic evaluation should be maintained when symptoms arise, including nonspecific clinical manifestations that could potentially indicate underlying malignancy (160, 165–167).

### 3.10 Limitations

This narrative review has several limitations that should be made explicit so that readers do not over-interpret the synthesis presented above.

#### 3.10.1 Limitations of the underlying human evidence base

With the exception of tesamorelin, which has been evaluated in randomized controlled trials within an approved clinical indication, the available human evidence for GH–IGF-1-axis performance-enhancing peptides consists predominantly of phase I pharmacokinetic and pharmacodynamic studies in healthy adults, small mechanistic endocrine studies, or paediatric GH-deficiency cohorts. Reported outcomes are typically restricted to acute hormone secretion or surrogate endocrine endpoints rather than to performance- or body-composition outcomes relevant to off-label users. Follow-up is short, sample sizes are small, populations are selected, performance-related endpoints are largely absent, and standardized exposure verification is not feasible in self-administering populations. Formal risk-of-bias scoring was therefore not performed; instead, evidentiary weight is conveyed at compound level through the stratification described in Section 3.5 and the Tier column of **Table 1**.

#### 3.10.2 Compound-level asymmetry of the evidence

The evidentiary basis differs by an order of magnitude across the compounds reviewed, ranging from regulatory-grade RCT data (tesamorelin, tier A), through phase I/II human studies without controlled efficacy data for performance-related endpoints (most GHRH analogues and ghrelin-mimetic secretagogues, tier B), to compounds with only limited or indirect human data (AOD9604, tier B/C), and finally to compounds for which no peer-reviewed human studies exist at all (CJC-1295 without DAC, PEG-MGF, IGF-1 LR3, tier D). Mechanistic plausibility for tier-D compounds does not constitute clinical evidence and should not be interpreted as such; readers and clinicians should explicitly consider this gradient when contextualising patient reports or laboratory findings.

#### 3.10.2 Methodological limitations of this review

This synthesis is a narrative review and does not constitute a systematic review. The literature search, while comprehensive within the defined scope, was not conducted against a pre-registered protocol, did not follow PRISMA reporting standards, and did not incorporate a formal risk-of-bias appraisal of individual included studies. Compound selection was driven by the agents most frequently encountered in clinical practice and most prominently described in unregulated user platforms, rather than by an exhaustive bibliographic enumeration. Residual selection bias inherent to narrative reviews therefore cannot be excluded, and the synthesis should be regarded as a structured clinical-orientation framework rather than as a definitive evidence assessment. SANRA-based self-assessment of reporting is provided in **Supplementary Table 1**.

#### 3.10.3 Limitations of the grey-literature mapping

Forum- and vendor-derived dosing protocols are presented in this review explicitly as anthropological and behavioural descriptors of unregulated self-administration patterns, not as clinical guidance. These sources are not peer-reviewed, are temporally unstable (protocols evolve and migrate across platforms over time), are subject to selection bias inherent to public-facing online communities, and cannot be independently verified for product identity, purity, or actual user behaviour. Their inclusion serves to characterise the magnitude and structure of the disconnect between real-world off-label use and the available clinical evidence base, and must not be read as endorsement of, validation of, or a basis for any specific dosing recommendation.

#### 3.10.4 Generalizability and the translation gap

Where controlled human data exist, they were generated in regulated clinical settings, in defined populations, using pharmaceutical-grade compounds at controlled doses, and with predefined endpoints. Off-label users typically self-administer compounds of uncertain identity and purity, in higher and more variable doses, frequently in stacked combinations with anabolic-androgenic steroids and other performance-enhancing agents, and over time-frames that substantially exceed the duration of available trials. The applicability of trial-derived effect estimates and safety profiles to this real-world population is therefore inherently limited, and the clinical recommendations summarised in **Figure 3** and Section 3.6 should be interpreted as expert-opinion–based pragmatic guidance rather than as evidence-based protocols.

## 4 Conclusions

Across the GH–IGF-1-axis peptides reviewed here, the clinically actionable signal is not compound-specific efficacy but a recurring pattern of class-level endocrine disruption: somatotropic activation with chronic IGF-1 elevation, ghrelin-mimetic prolactin–cortisol cross-talk for the GHRP subset, dysglycaemia, fluid-retention syndromes, and a biologically plausible but unproven mitogenic concern. These signals tend to dominate the clinical presentation regardless of which exact peptide a patient reports, and frequently arise within broader polypharmacy that includes anabolic-androgenic steroids and other performance-enhancing agents - making attribution to a single compound unreliable in real-world practice.

The practical implication for clinicians is therefore not to validate or optimise these regimens but to treat the patient encounter as an exposure-history and symptom-domain problem. Section 3.6 and **Figure 3** operationalise this approach through a non-judgmental disclosure interview, structured triage of dominant symptom domains, and explicit red-flag escalation criteria. With the singular exception of tesamorelin within its approved HIV-lipodystrophy indication, none of these compounds carry regulatory approval for performance- or appearance-related use, and tesamorelin trial data should not be extrapolated to hypertrophy or athletic indications. Research priorities differ markedly by evidentiary tier (Section 3.5): for tier-A/B compounds, the limiting factor is controlled trials with clinically meaningful endpoints, longer follow-up, and standardised exposure verification; for tier-C/D compounds, even basic safety pharmacology in humans is absent, and any clinical claim should be received with corresponding skepticism.
