# Supplementary material for: The emerging landscape of performance-enhancing peptides modulating GH-IGF1 axis: bridging the gap between clinical evidence and patient self-administration
Source: Front Endocrinol (Lausanne). 2026 Jun 18;17:1822475. doi: 10.3389/fendo.2026.1822475 (PMC13322892; doi:10.3389/fendo.2026.1822475)
Supplement: Supplementary file 1 [file Table1.docx]

**Supplementary Table S1.**

SANRA-based self-assessment of narrative review quality across the six SANRA domains.

| **SANRA domain** | **Score (0–2)** | **Justification** | **Manuscript location** |
| --- | --- | --- | --- |
| 1. Justification of the article's importance for the readership | **2** | The Introduction establishes the rising prevalence of off-label PEP use (Eurobarometer 2017; cross-European OTC prevalence 10.4% in 2023) and the clinician knowledge gap regarding GH–IGF-1-axis peptides outside formal medical supervision. Aimed explicitly at endocrinologists and other clinicians who increasingly encounter these patients. | Introduction |
| 2. Statement of concrete aims or formulation of questions | **2** | Three concrete aims are stated at the end of the Introduction: (I) map the main GH–IGF-1-modulating peptides used off-label and summarise PK/PD and human evidence; (II) synthesise endocrine-metabolic adverse effects relevant to clinical encounters; (III) compare clinical evidence with patient self-administration protocols and translate this into counselling and risk interpretation. | Introduction, final paragraph |
| 3. Description of the literature search | **1** | The Methods section reports the databases searched (PubMed, Google Scholar, Cochrane Library), time window (January 1989 – January 2026), language restriction (English), search terms at compound and class level, explicit compound-selection criteria (I/II/III), and a separate, transparently described mapping of grey-literature sources. The narrative-review design is justified because the heterogeneity of compounds, populations, endpoints, and study designs makes formal quantitative synthesis inappropriate. This item was rated as partially fulfilled because, despite a detailed narrative description, the search process is not documented with PRISMA-style procedural granularity (e.g. explicit screening flow), and the remaining gap was judged insufficient for a full score. | Section 2. (Literature Search Strategy) |
| 4. Referencing | **2** | Claims are supported by peer-reviewed primary literature and authoritative reviews. Peer-reviewed sources are clearly differentiated from grey literature throughout the manuscript, including in Tables 1–3 and the new Section 3.5. Compounds without human evidence (CJC-1295 without DAC, PEG-MGF, IGF-1 LR3) are explicitly flagged as such and not presented as evidence-supported. | Throughout; explicit stratification in Section 3.5 (Evidence stratification across compounds) |
| 5. Scientific reasoning | **2** | The discussion explicitly distinguishes (a) demonstrated endocrine effects, (b) unproven performance and hypertrophy claims, (c) intrinsic uncertainty in unregulated markets (product identity, purity, batch variability), and (d) potential harms with sparse long-term safety data. Limitations are addressed in the dedicated Section 3.10 and again in the Discussion. Oncologic risk is framed as biologically plausible but not causally demonstrated (Section 3.9). | Sections 3.5, 3.6, 3.7, 3.9, 3.10 and Conclusions |
| 6. Appropriate presentation of data | **2** | Tables and figures are used to summarise multidimensional information without spurious quantitative claims: Table 1 (per-compound mechanism, PK, safety signals), Table 2 (dosing patterns from peer-reviewed and grey-literature sources, with strong framing as anthropological/behavioural data), Table 3 (cross-class endocrine signals, qualitative descriptors), Figures 1 and 2 (mechanistic schemata), Figure 3 (clinical algorithm), Supplementary Table S1 (detailed step-wise rationale and per-step literature support for Figure 3). No statistical inference is presented because the design is narrative. | Tables 1–3, Figures 1–3, Supplementary Table S1 |
| **Total (max 12)** | **11** | Overall SANRA score: 11/12, reflecting high reporting quality across all domains with a moderate score for the description of the literature search (item 3). | |

**Notes.** Self-assessment performed by the authors using the SANRA tool (Baethge C, Goldbeck-Wood S, Mertens S. SANRA — a scale for the quality assessment of narrative review articles. Res Integr Peer Rev. 2019;4:5). Scores reflect author judgement and are not externally adjudicated. Each domain is rated 0 (not addressed), 1 (partially addressed), or 2 (fully addressed); maximum score 12.
