# Supplementary material for: The emerging landscape of performance-enhancing peptides modulating GH-IGF1 axis: bridging the gap between clinical evidence and patient self-administration
Source: Front Endocrinol (Lausanne). 2026 Jun 18;17:1822475. doi: 10.3389/fendo.2026.1822475 (PMC13322892; doi:10.3389/fendo.2026.1822475)
Supplement: Supplementary file 2 [file Table2.docx]

# Supplementary Table S2.

**Detailed clinical algorithm for the assessment of patients with suspected use of GH–IGF-1-axis performance-enhancing peptides (PEPs).**

This table is the detailed companion to Figure 3 in the main manuscript. It expands each of the four sequential steps and three triage outcomes presented in Figure 3 into the eight bedside steps used in clinical practice, and provides the rationale for each step. The intent is to preserve the evidentiary backbone of the algorithm in supplementary form so that Figure 3 can be read at a glance while clinicians who wish to review the underlying reasoning can consult this table.

| **Step** | **Clinical focus** | **Key actions** | **Rationale** |
| --- | --- | --- | --- |
| **1** | **Create conditions for disclosure** | • Use non-judgmental, curiosity-based questions (e.g. “Many people use supplements or research compounds… Have you tried anything like peptides, growth hormone, or IGF-1 products?”).  • Ask specifically about product names, source (pharmacy vs research chemical), route (SC, IM, oral), duration, and stacking with AAS, thyroid hormones, stimulants, insulin-sensitizers. | Shame, fear of stigma, and uncertainty about legality often lead patients to under-report or mislabel PEP use; without explicit, neutral questioning, exposure may remain hidden and laboratory abnormalities be misattributed. |
| **2** | **Clarify exposure pattern** | • Document compounds (if known), typical/maximum doses, injection frequency, cycle length, off-periods, and approximate timing of last use.  • Ask about co-use of multiple PEPs (GHRH analogue + GHRP + IGF-1 analogue) and concurrent AAS cycles. | Many endocrine abnormalities reflect cumulative or overlapping effects of several agents; timing relative to the last dose influences interpretation of IGF-1, cortisol, and prolactin. |
| **3** | **Identify dominant symptom domain** | Screen for:  • Fluid retention / soft-tissue symptoms (edema, paresthesias, arthralgia/myalgia, carpal-tunnel-like symptoms).  • Metabolic symptoms (polyuria, polydipsia, weight gain/loss, fatigue).  • Lactotropic / corticotropic symptoms (galactorrhoea, sexual dysfunction, low libido, mood/sleep disturbance).  • Local / infectious issues (recurrent injection-site inflammation, abscess, fever).  • Neurologic / visual symptoms (headache, diplopia, field defects). | Grouping symptoms into phenotypic clusters (GH/IGF-1 overactivation, dysglycaemia, prolactin–cortisol cross-talk, infection) helps guide targeted testing and risk stratification even when the exact compound identity is uncertain. |
| **4** | **Baseline and targeted laboratory evaluation** | • Minimum panel: fasting glucose or HbA1c, electrolytes, creatinine/eGFR, liver enzymes, lipid profile, full blood count, age-adjusted IGF-1.  • Add targeted tests based on phenotype:  – Prolactin and morning cortisol ± ACTH if lactotropic / corticotropic symptoms.  – TSH and free T4 if weight change, fatigue, or AAS co-use.  – CRP / WBC if infection suspected. | IGF-1 provides more stable information than random GH, but is modulated by nutrition, liver function, thyroid status, and acute illness; concurrent derangements (e.g. dyslipidaemia from AAS) may modify overall risk. |
| **5** | **Interpret endocrine patterns cautiously** | • Avoid overinterpreting single random GH measurements; rely on IGF-1 and clinical context.  • For mild prolactin or cortisol elevations, consider PEP-related pituitary stimulation and repeat testing after a wash-out period before proceeding to imaging.  • Recognise that GHRPs can acutely raise cortisol and prolactin, whereas GHRH analogues and IGF-1 analogues typically do not. | Without considering drug effects, mild hyperprolactinaemia or borderline hypercortisolemia can trigger unnecessary MRI or dynamic testing; conversely, assuming “benign” drug effects may delay investigation if red flags are present. |
| **6** | **Screen for red flags and need for urgent escalation** | Immediate escalation (emergency or urgent specialty review) if:  • Rapidly progressive dyspnoea or orthopnoea with marked edema, chest pain, or suspected heart failure / thromboembolism.  • Severe hyperglycaemia (e.g. symptomatic or markedly elevated glucose), ketoacidosis features, or hyperosmolar state.  • High fever or systemic toxicity with injection-site cellulitis / abscess.  • Severe headache, visual field defects, ophthalmoplegia, or altered consciousness.  • Suspected sepsis or necrotising soft-tissue infection. | PEP use may coexist with AAS and other agents that increase thrombotic, cardiometabolic, or infectious risk; red-flag symptoms should override assumptions that issues are “just from peptides”. |
| **7** | **Non-urgent endocrine referral and imaging** | Consider pituitary imaging and formal endocrine referral if:  • IGF-1 remains markedly elevated off-cycle and other causes are excluded.  • Persistent, unexplained hyperprolactinaemia or cortisol excess after documented PEP cessation.  • Progressive neurologic or visual symptoms even if drug effects are suspected. | A period of abstinence with repeat labs can unmask persistent pathology; however, deferring imaging is inappropriate when there are structural red flags or significant, persistent biochemical abnormalities. |
| **8** | **Counselling and follow-up** | • Explain what is known vs theoretical: clear separation between demonstrated endocrine effects (IGF-1 elevation, dysglycaemia, prolactin / cortisol changes, fluid retention) and unproven performance or hypertrophy claims.  • Discuss uncertainty regarding product identity, sterility, and long-term oncologic risk with chronic GH / IGF-1 stimulation.  • Offer harm-reduction–oriented follow-up (monitoring labs, supporting cessation, addressing AAS and other co-exposures). | Framing PEPs as unregulated, unapproved agents with uneven evidence helps avoid implicit endorsement while maintaining rapport; repeated contact improves opportunities for full disclosure and risk modification. |

**Abbreviations.** AAS, anabolic-androgenic steroids; ACTH, adrenocorticotropic hormone; CRP, C-reactive protein; eGFR, estimated glomerular filtration rate; GH, growth hormone; GHRH, growth hormone–releasing hormone; GHRP, growth hormone–releasing peptide; HbA1c, glycated haemoglobin; IGF-1, insulin-like growth factor 1; IM, intramuscular; MRI, magnetic resonance imaging; PEP, performance-enhancing peptide; SC, subcutaneous; T4, thyroxine; TSH, thyroid-stimulating hormone; WBC, white blood cell count.
